# Impact of Service User Video Presentations on Explicit and Implicit Stigma toward Mental Illness among Medical Students in Nepal: A Randomized Controlled Trial

**DOI:** 10.3390/ijerph18042143

**Published:** 2021-02-22

**Authors:** Cori L. Tergesen, Dristy Gurung, Saraswati Dhungana, Ajay Risal, Prem Basel, Dipesh Tamrakar, Archana Amatya, Lawrence P. Park, Brandon A. Kohrt

**Affiliations:** 1 Department of Psychology, DePaul University, Chicago, IL 60604, USA; ctergese@depaul.edu; 2 Duke Global Health Institute, Duke University, Durham, NC 27708, USA; larry.park@duke.edu; 3 Transcultural Psychosocial Organization Nepal, Baluwatar, Kathmandu, Nepal; dristy.1.gurung@kcl.ac.uk; 4 Institute of Psychiatry, Psychology, and Neuroscience, King’s College London, London SE5 8AF, UK; 5 Institute of Medicine, Tribhuvan University, Kathmandu, Nepal; iomsaras@gmail.com (S.D.); prembasel11@gmail.com (P.B.); 6 Department of Psychiatry, Kathmandu University School of Medical Sciences, Dhulikhel, Nepal; drajayrisal@gmail.com (A.R.); bob.dipesh@gmail.com (D.T.); 7 Save the Children, Kathmandu, Nepal; docarchana2013@gmail.com; 8 Infectious Disease Division, Department of Medicine, Duke University Medical Center, Durham, NC 27708, USA; 9 Division of Global Mental Health, Department of Psychiatry, George Washington University, Washington, DC 20037, USA

**Keywords:** attitudes, depression, developing countries, medical education, mental health, psychosis, service users, stigma

## Abstract

This study evaluated the impact of didactic videos and service user testimonial videos on mental illness stigma among medical students. Two randomized controlled trials were conducted in Nepal. Study 1 examined stigma reduction for depression. Study 2 examined depression and psychosis. Participants were Nepali medical students (Study 1: *n* = 94, Study 2: *n* = 213) randomized to three conditions: a didactic video based on the mental health Gap Action Programme (mhGAP), a service user video about living with mental illness, or a control condition with no videos. In Study 1, videos only addressed depression. In Study 2, videos addressed depression and psychosis. In Study 1, both didactic and service user videos reduced stigma compared to the control. In Study 2 (depression and psychosis), there were no differences among the three arms. When comparing Study 1 and 2, there was greater stigma reduction in the service user video arm with only depression versus service user videos describing depression and psychosis. In summary, didactic and service user videos were associated with decreased stigma when content addressed only depression. However, no stigma reduction was seen when including depression and psychosis. This calls for considering different strategies to address stigma based on types of mental illnesses. ClinicalTrials.gov identifier: NCT03231761.

## 1. Introduction

Stigma towards people living with mental illness impedes the provision of quality mental health services [1]. Stigma originates from beliefs and attitudes that have been identified among healthcare professionals as early as medical school [2,3]. Stigma against persons with mental illness is also common in low- and middle-income countries (LMICs) [4]. The majority of medical students in a study in India held a negative view of depression, and they did not consider it to be a real illness [5]. In a comparative study, depression was found to be more stigmatized by Sri Lankan medical students than among medical students in the United Kingdom [6]. In both high-income countries (HICs) and LMICs, there is conflicting evidence about whether medical school education improves or worsens attitudes towards mental illness. After completing a psychiatry rotation, student attitudes toward mental illness improved in a few studies [7,8,9], but they stayed the same or worsened in others [5,10,11]. When comparing the attitudes of non-clinical hospital staff versus clinicians, staff held more positive views of people living with mental illness [12]. Taken together, findings from these studies suggest that formal medical education fails to reliably improve attitudes towards mental illness, and, in some cases, it may increase stigma [5,10,12]. There is a need for research to evaluate interventions, especially in LMICs, to reduce mental health-related stigma among medical students [13,14].

There are two popular models for changing stigmatizing attitudes towards mental illness that have demonstrated effectiveness: education and contact [15]. The goal of educational approaches is to replace inaccurate stereotypes with true information about mental illness, whereas the goal of contact methods is to facilitate positive interactions of clinicians or clinical trainees with people living with mental illnesses [15]. When comparing both methods of improving attitudes towards people living with mental illness, contact interventions are demonstrated to be more effective than education among adults [16,17]. Contact methods aim to improve intergroup relations. Contact theory is based on the social psychological concept that when individuals spend time together with a common goal this fosters a shared group identity. This field of psychology was applied to the improvement of race relations during the civil rights movement in the US [18]. Contact theory can be applied to other groups as well, including people living with mental illness [18]. In addition, the contact can be either in person or through video recordings of testimonials. Studies conducted in HICs demonstrate that both in-person and indirect video contact methods reduce stigma towards mental illness [19,20], with in-person contact associated with a greater effect size [16]. In HICs, direct contact with mental health service users (persons utilizing mental health services) improved attitudes among healthcare students in nursing and pharmacy [19,21].

Medical schools should be targeted for stigma reduction interventions because students routinely express negative attitudes towards mental illness [2], and the current curriculum models might be increasing stigma [5,10,12]. With regard to medical students in HICs, contact with service users did not demonstrate attitude change [21,22]. A recent systematic review of interventions to reduce mental health-related stigma among medical and nursing students in LMICs identified nine studies from six countries [23]. A 4-day educational program in Nigeria for medical students and nurses that provided basic information about mental illness and treatments was shown to improve attitudes post-assessment on three out four subscales [24]. With regard to contact approaches, researchers demonstrated significant improvement in Turkish medical student attitudes on three out of seven items post-intervention and one out of seven items one month after contact with a person with schizophrenia accompanied by a video of people living with schizophrenia [25]. A randomized controlled trial (RCT) evaluated social contact methods (live service user contact vs. video recordings of service users) among medical students in Malaysia, revealing no differences between the two approaches [26]. Substantially greater efforts are needed to expand the evidence on what works in medical education stigma reduction.

Given extant work in HICs, using video-based approaches to social contact is a potentially impactful and cost-effective strategy [19]. Our objective was to compare two video-based approaches to stigma reduction among medical students in an LMIC. We selected Nepal because of growing efforts to expand mental health services [27] and prior documentation of stigma among Nepali clinicians [28]. In a three-arm trial, we compared the following conditions: 8 min didactic videos about a mental health condition; 8 min videos with service users presenting recovery testimonials; and a no-video condition. The goal was to examine immediate explicit and implicit stigma attitudes after video viewing. We evaluated these objectives through two studies. In Study 1, the didactic video and the service user video focused only on depression. In Study 2, the didactic arm included both depression and psychosis videos, and in the service user arm, medical students watched both a video of a person living with depression and a video of a person living with psychosis. We hypothesized that both video types would perform better than no video, and we hypothesized that the service user video would be more effective than the didactic video at reducing stigma.

## 2. Materials and Methods

### 2.1. Design and Setting

Nepal is a low-income country in South Asia where both stigma and inadequate pre-service training of health providers contribute to insufficient human resources for mental health services in primary healthcare settings [29,30]. Nepal is one of the many countries globally in which the World Health Organization (WHO) mental health Gap Action Programme Implementation Guide (mhGAP-IG) [31] is being used. The WHO developed the mhGAP-IG to educate non-specialized healthcare providers about delivering mental health services in low-resource settings. The mhGAP-IG has been evaluated as a component of a district mental health plan [27]. It is now the foundation of the government’s standard treatment protocol, which is being scaled up nationwide. Emerging research in Nepal on video-assisted mental health training demonstrated that pre-recorded didactic presentations based on the mhGAP-IG can be complementary tools to educate providers about mental health in rural, low-income settings [32]. There have also been growing efforts to reduce stigma through in-person social contact interventions embedded in mhGAP-IG training [33,34,35,36]. However, in-person service user approaches are resource intensive and potentially difficult to scale. Therefore, our goal was to use mhGAP-IG-based didactic videos and compare them with service user testimonial videos to evaluate the impact on stigma. We did this through 2 studies. 

Study 1 was a pilot RCT with a mixed-methods approach conducted in 2017 with medical students at Tribhuvan University’s Institute of Medicine (TU-IOM) in Kathmandu, Nepal. We employed a three-armed, parallel group RCT, and randomized participants to one of three conditions: a didactic video based on the mhGAP-IG module for depression; a service user testimonial video from a person who experienced depression; or a control featuring no video. For the mixed-methods component, we also included exploratory qualitative interviews with participants. At the time of this pilot, only the depression service user video had been completed, so the study was limited to depression.

Study 2 was a full RCT that evaluated the videos in a three-armed, parallel group RCT, in which medical students had an equal chance to be randomized to a service user testimonial video condition featuring lived experiences of depression and psychosis, didactic videos based on mhGAP-IG modules for depression and psychosis, or a control condition with no video. Data were collected from August 2017 through February 2018 at two medical universities in Nepal: TU-IOM and Kathmandu University School of Medicine (KUSMS) in Dhulikhel, Nepal.

Both studies were implemented in collaboration with Transcultural Psychosocial Organization Nepal (TPO), a nongovernmental organization engaged in research, services, and advocacy in Nepal.

### 2.2. Intervention Videos

Study 1 (depression only): The contents for all videos are presented in Table 1. All videos were 8 min in duration to have time matching between didactic and service user videos. The didactic video was adapted using existing mhGAP-IG materials from an ongoing study in Nepal: the Programme for Improving Mental Healthcare (PRIME) [37]. PRIME is a program used to evaluate the implementation of mental health in primary care in five countries, including Nepal [38]. Materials were already adapted and translated for Nepali healthcare settings in PRIME. We reduced the content delivered in the PRIME training package about depression to an 8 min video and collaborated with a local mhGAP-IG trainer who is a Nepali psychiatrist to narrate the video in Nepali. The content follows the mhGAP-IG depression module.

A service user video was produced in collaboration with the Reducing Stigma among Healthcare Providers (RESHAPE) initiative in Nepal. RESHAPE is a program in which service users treated through PRIME are trained in PhotoVoice participatory techniques to develop recovery narratives, which they use to co-facilitate mental health training for health workers [33,34,35,36]. The service user in this video was selected based on her previous success of co-facilitating mental health training in RESHAPE. The script for the video was based on the narrative prepared by the service user for her PhotoVoice testimonial. During video editing, the research team selected specific content from the testimonial that matched the content of the didactic video. Additional perspectives from a health worker who diagnosed the service user and a counselor who treated her were interwoven with the testimonial to produce the final 8 min video. The service user selected for the video was provided per diem compensation when attending PhotoVoice and health worker training. For her involvement in video production, she was paid as a consultant comparable to local journalist rates.

Study 2 (depression and psychosis): The didactic and service user testimonial videos developed for depression in Study 1 were also used in Study 2. In addition, didactic and service user videos were developed for psychosis. The psychosis didactic video, like the one about depression, was adapted from the mhGAP-IG psychosis module used in PRIME in Nepal. The same Nepali psychiatrist who narrated the depression video also narrated the psychosis one for consistency. The service user testimonial video about psychosis was delivered by a service user who received treatment through PRIME. Due to stigma and family shame, finding a service user who was willing to speak about psychosis on video was more challenging than finding someone for depression. A person was eventually identified, and she, alongside her family members and a counselor, produced the narrative video with the research team. Because both the 8 min psychosis and 8 min depression content were included, the total duration of videos in both the didactic and service user arms was 16 min in Study 2. The service users for both the depression and psychosis videos were women 20–30 years of age. 

### 2.3. Sample and Procedure

Study 1 (depression only): Participants were second- and third-year pre-clinical students in the Bachelor of Medicine, Bachelor of Surgery (MBBS) program at TU-IOM. The MBBS is a five-year undergraduate medical program. The native language of the participants was Nepali, but the MBBS program was taught in English. Participants were excluded from the study if they were younger than 18 years old, had finished their psychiatry rotation, or were international students who were not native Nepali speakers, because all videos were in Nepali. Because this was a pilot study, we did not conduct a power analysis to determine the sample size. We recruited one hundred students. During recruitment, the local collaborators at TU-IOM, the principal investigator, and the Nepali research assistant visited students in their classrooms, described the research activities, and gathered their contact information. During the recruitment sessions, information was delivered in both English and Nepali describing the nature of study and the intended goals. The researchers stated that participation in the study was voluntary and that there would be no negative consequences if they chose not to participate or if they wished to drop out of the study. The class representatives for each grade level assisted by verifying contact information when information was missing. Prior to scheduled data collection sessions, students on the contact list were stratified by grade level and then randomized to one of the conditions using a computer-generated random order. Students were informed that the study was about mental health knowledge, attitudes, and clinical practices. However, they were not explicitly told that this was about stigma reduction because of concern that this could have biased responses.

Study 2 (depression and psychosis): First- and second-year medical students in the MBBS programs at TU-IOM and KUSMS participated in this study. The same exclusionary criteria applied as in Study 1. The sample size was determined by conducting a power analysis of preliminary results from another study in Nepal that shared the primary outcome of this study. The calculations estimated that 67 participants would be needed in each condition, or 201 overall, to detect a statistically significant difference in social distance scores (see Outcome Measures below) between the didactic and control conditions (α = 0.05, power of 0.80). To account for potential dropouts, the minimum target was 213 participants. Recruitment took place for the duration of data collection and ended when the sample size was achieved. As with Study 1, during classroom visits, research activities were described, and contact information was gathered from all of the eligible participants. As with Study 1, eligible participants were stratified, randomized, and blinded to the specific focus on stigma in order to minimize potential response biases.

### 2.4. Outcome Measures

The forms and assessments used during data collection were forward and back translated from English to Nepali. Culturally specific Nepali idioms were utilized when applicable [39,40,41]. All quantitative data collection was conducted in Nepali. The primary outcome was explicit attitudes, i.e., those attitudes that a person actively endorses, on the Social Distance Scale (SDS), with responses on a Likert scale with items referring to willingness to interact with people who have mental illness. Twelve items with options from 1 to 6 produce a range of 12 to 72, with higher scores indicating greater social distance-related stigma. The SDS was adapted from a version in the Stigma in Global Context—Mental Health Study [42,43], and it is a widely used measure to assess willingness to interact with persons from a specific stigmatized group [44,45]. The internal consistency of SDS in Nepal is high (α = 0.80) [34]. 

Implicit bias evaluated with the Implicit Association Test (IAT) [46] was a secondary outcome. Whereas explicit attitudes are considered consciously endorsed beliefs and preferences, implicit attitudes refer to associations that an individual may not be aware of but influence perception and behavior. Implicit attitudes have been linked to clinical decision making [47]. Implicit bias is operationalized in IATs as *d*-scores, in which scores farther from zero in either negative or positive directions represent greater implicit preference for physical or mental illnesses on specific attributes. Two Nepali IATs were developed in Nepali: one was an adaptation of physical vs. mental illness on harmlessness vs. harmfulness. In the harmfulness IAT, a positive *d*-score refers to greater implicit of association of mental illness with harmfulness. A second was locally developed as physical vs. mental illness on burdenless vs. burdensome, because of the cultural perception that mental illness is associated with greater burden on the health system, societies, and family [35]. In the burdensome IAT, a positive *d*-score reflects an association of mental illness with burdensomeness. The *d*-scores were calculated using a recommended algorithm [48]. 

Another secondary outcome was explicit attitudes towards two patient vignettes, which were previously adapted for use in Nepal [49,50]. One vignette described symptoms of depression and the other described symptoms of psychosis. Participants used a five-point Likert scales to describe how much they agreed with following statements: (a) being around [a person with depression or psychosis] in public would make me feel unsafe, and (b) someone like [a person with depression or psychosis] would do something violent to hurt others around him. Lower scores indicate higher agreement and thus greater stigma. 

For a diagnostic accuracy secondary outcome, we also used the adapted Nepali written vignette of a person experiencing depression and a person experiencing psychosis. In Study 1, open-ended diagnostic impression responses were coded as correct or incorrect by the principal investigator. In Study 2, participants also had to correctly choose appropriate treatments in a multiple-choice question for each vignette. There were at total of four correct and four incorrect options with a possible total score ranging from −4 to +4.

For Study 1, qualitative interviews were conducted by the first author in English; participants reported that this was acceptable because the language of the medical school instruction was English. We conducted one-hour, in-depth interviews with a subset of the participants in each arm. The interview questions were created collaboratively by Nepali and American researchers. The purpose of these interviews in the pilot study was to test a semi-structured interview guide, begin development of a codebook, and identify salient themes pertaining to four primary topics of interest. The four topics were: the best ways to teach students about mental health, the prevailing attitudes towards mental health, the reasons why students do not specialize in psychiatry, and student evaluations of the videos.

### 2.5. Data Collection and Management

The intervention was a single session that lasted approximately one hour for participants to provide informed consent, complete a demographic form, watch videos relevant to their study arm, complete the assessments, and be debriefed. Participants in either of the video groups stayed for the full hour, but participants in the control condition who did not watch the video finished in about 50 min. In Study 2, which included two 8 min videos with diagnostic accuracy for both conditions, the protocol required an additional 15 min. Survey data were collected using SurveyCTO (Ver 2.30, Dobility, Inc., Cambridge, MA, USA), an android application, on tablets (www.surveycto.com/accessed on: 19 June 2017). In Study 1, after completion of the video session, participants were invited to participate in the optional qualitative interview to be held within one week. 

### 2.6. Ethics and Human Subjects’ Protection

At the beginning of each study session, a verbal explanation about the study and the consent form were given in Nepali by the research assistant, and written informed consent was gathered from participants in a forward and back translated document presented in Nepali. During debriefing, participants were informed that they could still withdraw from the study by contacting the research staff without penalty. A mental health resource list was emailed to participants if they had further questions, concerns, or interest in mental health services. No participants self-disclosed experiencing distress during the procedure, so none were referred for mental healthcare during the study. Participants were not compensated for their participation in the research activities. The procedures for this study were approved by the ethical review boards at Duke University (E0078), TU-IOM (380), KUSMS, and the Nepal Health Research Council (146/2017) prior to data collection. All data were stored on an encrypted server and validated by a second investigator before performing analyses. 

### 2.7. Data Analysis 

Descriptive statistics summarized the characteristics of the participants. The primary inferential analyses evaluated differences in attitudes and knowledge by comparing intervention groups. Differences in attitudes and knowledge between study conditions were analyzed using linear and logistic regression. The primary comparisons were between (a) both interventions compared to the control and (b) the two intervention groups to each other. Pearson correlation was used to identify relationships between implicit and explicit attitudes as well as correlations between attitudes and knowledge. The implicit and explicit correlation was done to test if reported and unconscious beliefs were related. The other correlation tested if greater knowledge was associated with more positive attitudes. Analyses using IAT data only included frequent computer users. A subsequent regression model was adjusted for personal experience with mental illness to determine the association between the intervention videos and the primary outcome. We conducted an exploratory analysis comparing findings from Study 1 (depression only) and Study 2 (depression and psychosis). All analyses were performed in STATA software v. 15.0 [51] with two-tailed tests using a significance level of 0.05. 

For the qualitative component, interviews were audio recorded and transcribed for analysis. Data management and coding were facilitated using QSR NVivo 11 software [52]. Data analysis was guided using a content analysis strategy. First, the interviews were transcribed, and then two independent reviewers wrote memos for each of the transcripts to identify salient themes and potential codes. A codebook was created using inductive and deductive sources of information to generate major and minor categories of themes. Each code under a theme had a unique definition and inclusion/exclusion criteria. Both researchers applied the codes to each transcript independently, discussed discrepancies until consensus was reached, and iteratively modified the codebook after each discussion. Codes were merged and sub-categorized into different themes for optimal organization and fit. All the transcripts were coded through this technique. The principal investigator wrote thick descriptions of the emergent themes and selected representative quotes. Presentation of the exploratory results described codebook themes that addressed the four topics of interest with exemplary quotes.

## 3. Results

### 3.1. Study 1 (Depression only)

#### 3.1.1. Study 1 Sample Description

**Figure 1:**
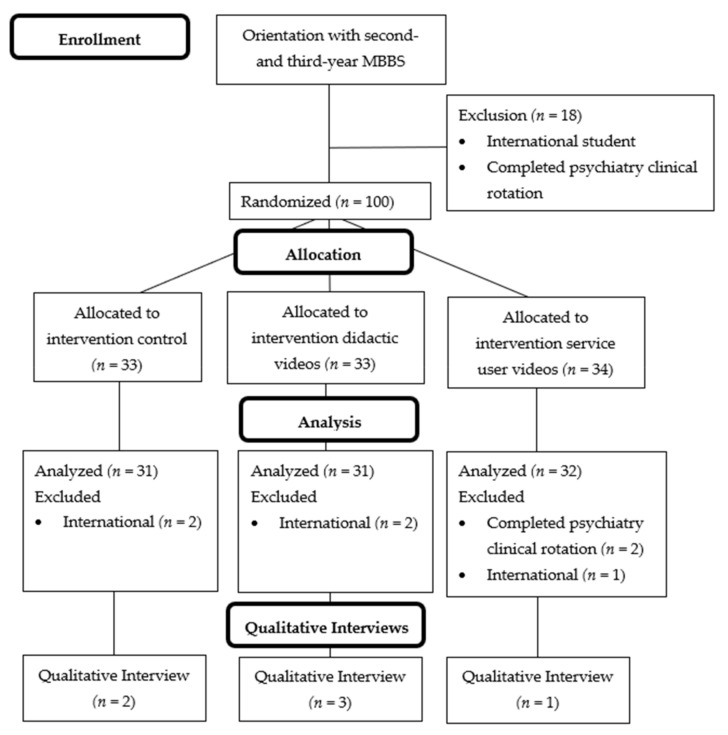
CONSORT flow diagram for research procedures in Study 1 (depression only). Note: in the service user video study arm, one participant excluded at the analysis phase met both the psychiatry rotation and international exclusion criteria. Abbreviations: MBBS, Bachelors of Medicine, Bachelors of Surgery.

**Figure 2:**
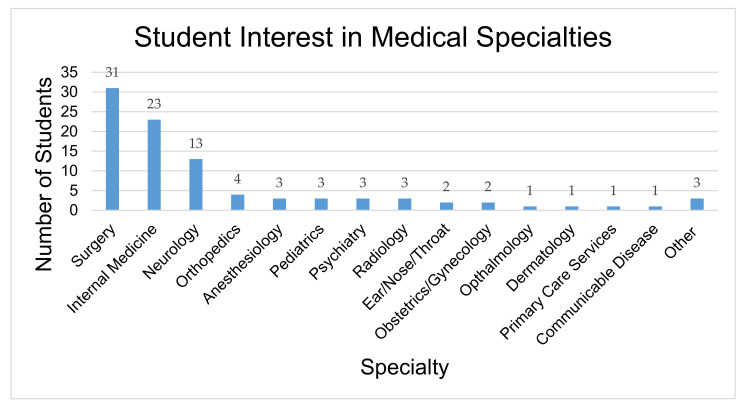
Second- and third-year medical students expressed interest in specific medical specialties (*n* = 94). Answers were selected from a multiple-choice question of common specialties offered in Nepali medical school.

After review of 2nd and 3rd year MBBS student rosters, 18 students were excluded prior to randomization because of being international students not speaking Nepali or having already completed their psychiatry rotation. Among the remaining students, 100 were selected for randomization to one of the three arms. No potential participants refused to participate in this study. An additional six students were excluded at the time of analysis because information on their demographic forms revealed that they were international students whose native language was not Nepali or they had completed their clinical psychiatry rotation; this information had not been up to date in the class rosters at the time of randomization (Figure 1). One participant in the service user arm was excluded because of both being an international non-Nepali student and having completed a psychiatry rotation. Demographic characteristics of these participants are in Table 2. Of note, only three participants indicated that they were primarily interested psychiatry as a specialty (see Figure 2). Participants were randomized into one the three conditions: the control group with no video (*n* = 31, 33%), the didactic video group (*n* = 31, 33%), and the service user recovery testimonial video group (*n* = 32; 34%).

#### 3.1.2. Study 1 Outcomes

Explicit attitudes: A linear regression that predicted social distance based on intervention conditions found that participants in the didactic lecture (*M* = 33.32; *SD* = 9.57) and the service user testimonial (*M* = 30.13; *SD* = 9.16) had significantly lower SDS scores compared to the control group (*M* = 39.10; *SD* = 11.14) (*F*_2,91_ = 6.37, *p* = 0.003, *R*^2^ = 0.12) (Table 3). A second linear regression found that the difference between the two video groups regarding depression was not statistically significant (*F*_1,61_ = 1.55, *p* = 0.23, *R*^2^ = 0.02). 

Implicit attitudes: We analyzed IAT data for participants who self-identified as frequent computer users (*n* = 87, 93%) on the demographic form to avoid computer literacy bias. For the harmfulness attribute on the IAT, a linear regression (*F*_2,84_ = 1.19, *p* = 0.31, *R*^2^ = 0.03) showed that the didactic lecture (*M* = 0.07; *SD* = 0.33) and the service user testimony (*M* = 0.04; *SD* = 0.36) compared to the control condition (*M* = 0.15; *SD* = 0.32) did not significantly differ regarding implicit attitudes that people with mental illness were more harmful than people who have physical illness (Table 3). The difference between the video conditions was also not statistically significant (*F*_1,56_ = 0.88, *p* = 0.35, *R*^2^ = 0.02). A linear regression for the burdensomeness IAT (*F*_1,84_ = 0.88, *p* = 0.50, *R*^2^ = 0.02) found that the didactic lecture (*M* = 0.10; *SD* = 0.34) and the service user testimony (*M* = 0.17; *SD* = 0.29) did not significantly differ regarding implicit associations that people with mental illness were more burdensome than people with physical illness compared to the control group (*M* = 0.20; *SD* = 0.31) (Table 3). The two video groups were not statistically different from each other (*F*_1,56_ = 0.62, *p* = 0.43, *R*^2^ = 0.01).

Diagnostic accuracy: A logistic regression indicated that didactic (M = 84%; SD = 37%) and service user (M = 91%; SD = 30%) intervention groups did not significantly predict diagnostic accuracy compared to the control (M = 77%; SD = 43%) (X^2^(2) = 2.10, *p* = 0.35) (Table 3). Additionally, the service user and didactic videos did not significantly differ in terms of diagnostic accuracy (X^2^(1) = 0.65, *p* = 0.43).

#### 3.1.3. Study 1 Qualitative Findings

Due to limited time availability on the part of the researchers and students as well as the exploratory nature of the interviews, only six participants completed interviews. Qualitative results were analyzed from a subset of six students, two women and four men in their third year, who participated in in-depth interviews.

Ideal format for mental health education: Based on preexisting opinions regarding mental health education, we asked students to describe the best teaching method to learn about mental illness during their MBBS. Four students recommended hands-on experience outside of the classroom. Three of the students were eager to interact with service users outside of the classroom to acquire this type of experience. One student explained that meeting patients in person can bring about change within students to reduce treatment bias against mental health patients. This student recommended that the exposure should take place in the first year of school just after enrollment because it will leave a lasting impression on students for the rest of their careers.

Stigma towards persons living with mental illness: All the participants had observed some level of stigma towards people with mental illness before. They universally agreed that their communities held stigmatizing views and ostracized people living with mental illness. “Madness” (Nepali: *paagal* or *baulaahaa*) was a stigmatizing term used by three of the participants to describe someone who has mental illness in society. One student described madness as a situation where *“you’re not caring for yourself, and you harm other people. [It’s] when you are completely useless to society and evil towards human beings. It is harmful to humanity”*.

Participants held mixed opinions about whether their peers and faculty held negative views of mental health patients, particularly in reference to the association of mental illness with violence. Even though three of the participants stated that they did not believe doctors treated mental health patients differently than patients with physical illnesses, they still described examples of providers, or even themselves, stigmatizing individuals with mental health problems. In one instance, a student said that it was not appropriate to marry someone with psychosis because that person is unpredictable and dangerous. Another student recommended that dangerous, mad people be sent to “the asylum”. A third student proceeded to describe people living with mental illness as dangerous: *“I would like to work alongside [someone with depression], but I have a fifty percent chance that he might attack me. Or if he is depressed, he might not feel good, and he might do something to me.”* One student strongly believed that his peers and faculty held biases against people living with mental illness. When someone is feeling sad or down, the participant said fellow students reacted, *“You are being psycho or you are being mad. […] Even if you have cancer, people take it normally, but if you have a mental health problem, people take it seriously”*.

When students were asked why psychiatry was not a popular medical specialty, they identified stigma, familial pressure, faculty disregard, and low salary as barriers dissuading students from specializing in psychiatry. Two participants listed stigma as the primary force deterring students away from the field. One student said the main reason for a lack of interest is because society does not view psychiatrists as real doctors, and therefore, family would typically not allow them to become psychiatrists. Another student described how students become confused during their psychiatry rotation because their desire to help patients conflicts with information from teachers, seniors, and the community that psychiatry is unimportant. Two participants reported that faculty in the medical school promulgate negative views among the students; each separately described an instance when they heard a professor say, *“The difference between a psychiatrist and psychiatric patient is only the coat.”* Another participant stated that low salary was another factor dissuading students from specializing in psychiatry.

Perspectives on video content: Among the qualitative interview participants, three students viewed the didactic lecture video and one viewed the service user testimonial. Those who viewed the didactic lecture all agreed that the video was informative and useful for educational purposes. All three participants recommended that the video should include some type of illustration, picture, or story to increase viewer engagement. One student wanted the didactic video to include a recovery story of a patient who underwent counseling. He believed that recovery stories could change perceptions and reduce stigma about mental illness.

### 3.2. Study 2 (Depression and Psychosis)

#### 3.2.1. Study 2 Sample Description

**Figure 3:**
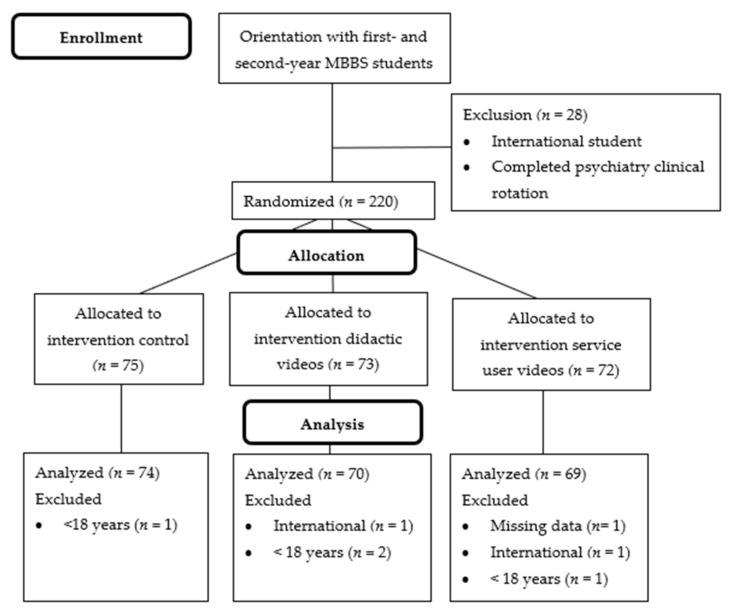
CONSORT participant flow diagram for research procedures in Study 2 (depression and psychosis). Abbreviations: MBBS—Bachelors of Medicine, Bachelors of Surgery.

For the second study, 248 students were enrolled in first- and second-year MBBS program across the two institutions participating. From roster, 28 students were excluded because of being international or having completed a psychiatry clinical rotation. The remaining 220 students were randomized; however, seven students declined to participate or were unavailable during data collection periods. Therefore, 213 participants were randomly allocated to the following arms: didactic video condition (*n* = 73), the service user video condition (*n* = 72), and the no video control condition (*n* = 75) (Figure 3). At the analysis phase, there were additional exclusions because of missing data or identification of exclusion criteria that was not recorded in the school registers. Participant characteristics for each condition are shown in Table 4.

#### 3.2.2. Study 2 Outcomes

Explicit attitude: Linear regressions were used to determine the effects of the video interventions on attitudes. In a linear regression model, neither the didactic videos nor service user testimonial videos predicted social distance compared to the control (*F*_2,210_ = 2.07, *p* = 0.13, *R*^2^ = 0.02) (Table 5). In the same model, there was no significant difference between the didactic and service user testimonial videos (*p* = 0.06). Therefore, the intervention videos had no effect on the primary outcome. A linear regression found that service user testimonials, but not didactic videos, predicted the degree to which participants agree that they would feel unsafe around a person who has depression in a public setting compared to the control (*F*_2,210_ = 3.74, *p* = 0.03, *R*^2^ = 0.03). When comparing the two video conditions to each other in this model, there was a significant difference (*p* = 0.02). However, neither of the intervention videos compared to the control predicted how much participants agreed that they would feel unsafe around someone who has psychosis (*F*_2,210_ = 0.40, *p* = 0.40, *R*^2^ = 0.01), and there was no significant difference between the video conditions (*p* = 0.42). It was found in another linear regression that service user testimonials, but not didactic videos, compared to the control predicted how much participants agree that people with depression would act violently towards others around them (*F*_2,210_ = 4.49, *p* = 0.01, *R*^2^ = 0.04). In the previous model, there was a significant difference between the service user testimonials and didactic videos (*p* = 0.02). When the mental illness was changed to psychosis, a linear regression model did not find that the intervention videos compared to the control predicted how much participants agreed that people with psychosis are violent (*F*_2,210_ = 2.73, *p* = 0.07, *R*^2^ = 0.04). The difference between video conditions in this model was not statistically different (*p* = 0.12).

Implicit attitudes: For analyses involving IAT data, only participants who identified as frequent computer users (*n* = 185) were included. Additional data were missing from each IAT (*n* = 3), so the final IAT analyses included fewer participants (*n* = 182). Neither the didactic videos nor the service user videos compared to the control were found to predict IAT scores for the harmfulness attribute in a linear regression (*F*_2,179_ = 0.37, *p* = 0.69, *R*^2^ < 0.01) (Table 5). There was also no difference between the intervention videos in this model (*p* = 0.40). Similarly, neither of the intervention videos compared to the control predicted IAT scores for the burdensomeness attribute (*F*_2,179_ = 0.42, *p* = 0.66, *R*^2^ < 0.01), and the didactic videos and service user testimonials were no different from each other either (*p* = 0.71).

Diagnostic accuracy: With regard to knowledge about mental illness, a logistic regression found that the didactic videos condition, but not the service user testimonials, compared to the control predicted accurate diagnosis of both depression and psychosis (*X*^2^(2) = 17.27, *p* < 0.001, *R*^2^ = 0.14) (Table 5). Both the service user testimonials and didactic videos compared to the control predicted treatment accuracy for depression in a linear regression (*F*_2,210_ = 6.40, *p* = 0.002, *R*^2^ = 0.06), and the difference between the video conditions was not significant (*p* = 0.31). Treatment accuracy for psychosis was also predicted by both video conditions compared to the control (*F*_2,210_ = 6.16, *p* = 0.003, *R*^2^ < 0.06), but the didactic and service user videos were not statistically different (*p* = 0.56).

Relationships between attitudes and knowledge: Pearson correlations were conducted to assess the relationship between explicit and implicit attitudes as well as knowledge of diagnosing and treating depression and psychosis (Table 6). Social distance scores, an outcome of explicit attitudes, were correlated with three out of four of the other explicit attitudinal outcomes: feeing unsafe in public near someone with depression (*r* = −0.23, *p* < 0.001), feeling unsafe in public near someone with psychosis (*r* = −0.30, *p* < 0.001), and belief that people with psychosis are violent (*r* = −0.18, *p* = 0.008). The direction of these correlations indicates that greater social distance is associated with the greater endorsement of stigmatizing views and attitudes towards mental illness. Both IATs for harmfulness and burdensomeness were positively associated such that participants who viewed mental illness as more dangerous than physical illness were also more likely to view people with mental illness as more burdensome to treat (*r* = 0.27, *p* < 0.001). Additionally, the IAT for burdensomeness was correlated with feeling unsafe in public near someone with psychosis (*r* = −0.19, *p* = 0.007), which indicates that the implicit attitude that mental illness is burdensome is associated with the fear of being around someone who has psychosis. 

Moderating Effect of Personal Experience with Mental Illness: To identify factors influencing the full RCT effects on social distance scores, a follow-up regression model was run. Personal experience with mental illness was found to be a significant predictor of social distance (*F*_1,178_ = 8.19, *p* = 0.005, *R*^2^ = 0.04) where social distance scores were 4.65 points higher among participants who did not have experience with mental illness. The regression model adjusted for personal experience with mental illness as an individual, family member, or close friend only included participants who definitively confirmed or denied experience (*n* = 178). With personal experience adjusting the model, a significant linear regression was found (*F*_3,178_ = 5.41, *p* = 0.001, *R*^2^ = 0.09), where the service user testimonial videos, but not the didactic videos, predicted social distance compared to the control (Table 7). After adjusting this model, there was also a significant difference between the two video conditions (*p* = 0.03). This model illustrates that the relationship between intervention condition and social distance scores was moderated by personal experience with mental illness such that the service user videos were associated with increased social distance compared to the control among people with no personal experience. Alternatively, personal experience with mental illness was a protective factor against increased social distance scores when exposed to the service user testimonial videos. 

### 3.3. Comparison between Study 1 (Depression only) and Study 2 (Depression and Psychosis)

**Figure 4:**
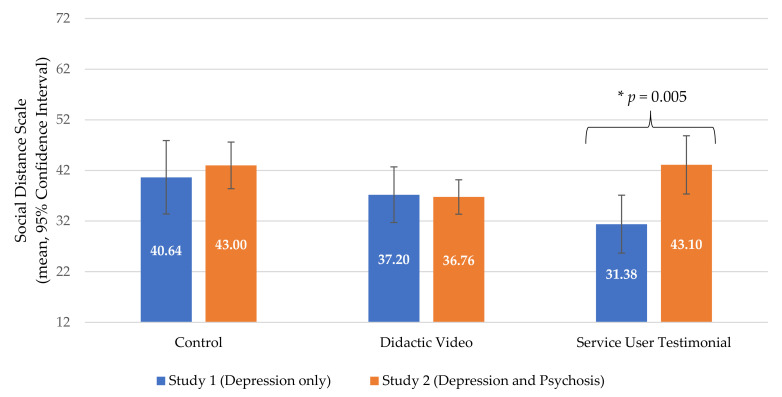
Social distance towards mental illness among second-year medical students at Tribhuvan University in Study 1 (depression only, *n* = 45) vs. second-year medical students at Tribhuvan University in Study 2 (depression and psychosis, *n* = 51).

The findings from Study 2 featuring depression and psychosis videos indicate that the service user testimonials did not change explicit attitudes on the primary outcome, which contradicts findings from Study 1. Secondary analyses were conducted to compare second-year MBBS participants from TU-IOM who participated in Study 1 (*n* = 45) and second-year MBBS participants from TU-IOM who participated in Study 2 (*n* = 51). These were unique participants. These two groups were similar demographically and educationally. The main difference between the trials was that Study 1 only included content about depression and Study 2 included content about depression and psychosis. Mean SDS scores were compared by intervention condition (Figure 4), and independent sample *t*-tests were conducted to compare Study 1 and Study 2. Among students in the control condition, there was no significant difference in social distance scores between Study 1 (*M* = 40.64, *SD* = 12.56) and Study 2 (*M* = 43.00, *SD* = 8.63) (*t*(28) = −0.61, *p* = 0.55). Similarly, there was no significant difference between social distance scores in Study 1 (*M* = 37.20, *SD* = 9.91) and Study 2 (*M* = 36.76, *SD* = 6.98) among students in the didactic video conditions (*t*(30) = 0.15, *p* = 0.89). However, in the service user testimonial groups, social distance scores were significantly higher in Study 2 (*M* = 43.06, *SD* = 11.56) than Study 1 (*M* = 31.38, *SD* = 10.73) (*t*(31) = −3.04, *p* = 0.005).

## 4. Discussion

These studies evaluated whether brief video-based didactic lectures or service user testimonials were associated with differences in explicit and implicit attitudes, as well as knowledge, and whether they were possible solutions to reduce stigma and increase knowledge about mental illness among medical students. Our results suggest potentially different patterns of immediate impact on attitudes based on the type of video (didactic vs. service user testimonial) and the specific mental illness. For depression, a small study with 94 participants suggests that an mhGAP-IG didactic video in Nepali and a service user recovery testimonial video were comparably beneficial for attitude and knowledge change. It is noteworthy that the magnitude of the change in social distance (SDS mean score) was greater in the service user depression video than in the didactic video, although this was not a statistically significant difference with a small sample size of approximately 30 participants per arm.

In contrast, in a study in which medical students viewed content for both depression and psychosis, we did not observe this pattern. Medical students in the didactic video arm and no video control arm were nearly indistinguishable in levels of explicit stigma (SDS mean score), and the service user depression and psychosis testimonial arm had a higher SDS score, although non-significant, compared to control and didactic arm participants. This raises the question of a potential interaction effect between the approach to immediate attitudinal changes and type of mental illness. In exploratory analyses with a subgroup of participants with similar demographics in Study 1 and Study 2, we found that depression service user videos performed well compared to other approaches. However, the combination of depression and psychosis across two service user videos did not show these gains. In contrast, a didactic video for depression only vs. didactic videos for depression and psychosis performed comparably on stigma outcomes in the subsample. The study was not designed for this specific comparison of how different types of mental illnesses moderate the effect of video-recorded approaches to social contact. That said, the findings raise important questions on what strategies should be pursued and evaluated going forward. 

These findings shed light on why interventions may contribute to differences in attitudinal outcomes. In two studies conducted in Canada with medical students, no differences were observed in attitudinal changes [21,22]. In one study, the service user was a person living with schizoaffective disorder, and in the second study, the service users included one person with medication-induced depression with psychotic symptoms and a second service user with narcolepsy and narcissistic personality disorder. Our finding regarding the combination of psychosis and depression-centered testimonials and similar studies with testimonials about psychosis may be related to the differential conceptualizations of these conditions. 

There is evidence that different mental illnesses might be stigmatized for different underlying reasons. For instance, people are more likely to view psychosis as a dangerous, uncontrollable, ingrained characteristic [53], whereas depression is considered to be self-regulated [54]. These negative perceptions lead to different forms and intensity of stigmatization. Because psychosis is viewed as more dangerous than depression [53], the testimonial video about psychosis might have elicited fears in participants, which in turn led to increased social distance towards people with mental illnesses. Within a community in India, people wanted greater social distance from persons with psychosis compared to those with depression [55].

Another interesting outcome of our studies was an exploratory analysis on the moderating effects of personal experience with mental illness as an individual, family member, or close friend. Personal experience appeared to be a protective factor against increased social distance when exposed to the service user testimonial describing psychosis, but students who lacked personal experience were susceptible to the potential unintended increase in stigma following exposure to depression and psychosis videos together. These findings support existing literature regarding the positive association between personal experience with mental illness and improved attitudes among medical students [11,56,57,58]. Even compared to psychiatry educational experiences during medical school, personal experience has been found to be a greater predictor of attitudes towards mental illness [58], which is likely why it was a moderating factor in this study. 

Our study also suggests that pairing depression and psychosis may unintentionally lead to increases in negative stereotypes about depression specifically. Exposure to the testimonial video about psychosis might have caused students to overgeneralize stigmatizing stereotypes from psychosis to depression. This is supported by the findings that participants in the service user condition compared to the didactic and control conditions were more likely to agree that they would feel uncomfortable around someone with depression and think that people with depression are violent. To avoid overgeneralizations, dispelling stereotypes should be a priority in stigma reduction interventions. 

This study’s intention was to develop a stigma reduction tool for medical training programs in LMICs, such as the mhGAP-IG, that could be scaled easily in a video format, but the differing results from the depression only study vs. the depression and psychosis study highlight the potential unintended generalization of negative attitudes when bundling mental illnesses into the same program. The current mhGAP-IG relies on the bundling method to concisely disseminate information and scale mental health services in low-resource settings. Based on the findings in this trial, the educational, bundled format of the mhGAP-IG might be inadvertently increasing stigma towards less stigmatized mental illnesses like depression, especially among trainees with limited personal experience with mental illness. 

Other initiatives in Nepal which have used in-person approaches to social contact for the mhGAP-IG have included persons living with depression, psychosis, alcohol use disorder, and epilepsy. Results from this in-person approach with multiple disorders have shown both immediate and long-term improvement in stigma [35]. This suggests that bundling conditions together is possible, but it may require in-person approaches as well as more extensive training as opposed to just a brief video session, as piloted in the current studies. Watching a testimonial video is a passive activity that does not engage the viewer in social contact like meeting a service user in person. As opposed to video testimonials, integrative training featuring in-person contact and group activities with service users might be a better method to improve attitudes. 

Regarding qualitative findings, interviews revealed that stigma towards mental illness was prevalent among students, faculty, and community members. Although half of the participants denied that stigma towards mental illness interferes with medical treatment, most of them still admitted that they or their peers would personally avoid people with mental health problems. Dangerousness was a common theme during the interviews. Three participants described mental health patients as dangerous, which supports existing literature that people living with mental illness are often stereotyped as “dangerous” [53]. In India, a country that shares cultural similarities with Nepal, 40.0% of the population perceived people with depression as dangerous [55]; in this study, dangerousness was the greatest predictor of social distance, so eliminating the perception of people with mental illness as dangerous is critical to improving public attitudes. In studies of formal education for student health professionals, training did not change perceptions of mental illness as dangerous [59,60,61]. It was suggested that interactions with the most severe cases of mental illness most likely occur during health education, and these interactions only reinforce negative stereotypes [20,53]. Other studies in South Asia suggest that the medical curricula may not reliably improve attitudes or increase interest in psychiatry [8,9,10]. Giasuddin and colleagues suggest that the quality of the psychiatry curricula, the location and perspective of the training, and the attitudes of teaching faculty might all increase stigma among students [10]. Therefore, more attention is needed to reduce stigma. 

Ultimately, our findings suggest that for severe mental illness, such as psychotic disorders, the inclusion of brief testimonial videos as a sole strategy is unlikely to be beneficial. As one interviewee pointed out, combatting negative stereotypes during contact with service users should include recovery stories to challenge existing beliefs. The results we achieved with in-person social contact with service users with psychosis in attitudinal change, including attitudes regarding violence, among healthcare workers in Nepal [35] were not replicated with this brief video exposure among medical students. Furthermore, there is evidence to support the integration of clinical postings with destigmatizing education and contact-based interactions. In a systematic review featuring studies in LMICs, both medical students and nurses demonstrated significant improvement in attitudes towards people living with mental illness when their respective clinical postings were augmented with didactic methods [62,63]. Videos featuring service users and didactic information could be used as supplemental tools during clinical postings in mental health training for medical students.

### Limitations

There were multiple limitations to this study. First, we did not conduct a power analysis to determine the sample size for Study 1 because it was designed as a pilot. The lack of a statistical difference in many analyses might be due to an underpowered study rather than the efficacy of the service user intervention. An underpowered study might also explain why the *r*^2^ values were low in the analyses that were significant. Additionally, despite the efforts to include culturally specific Nepali terms, it is possible that there might be validity issues with the IAT scores in this population because some students reported issues with Nepali vocabulary. Because the IAT is time sensitive, unfamiliar mental health terms might affect response times. 

We only qualitatively interviewed six participants in this pilot, so reported findings must be interpreted cautiously. With few participants, it was unlikely that we captured the depth of themes to fully address the research questions. The purpose of the qualitative interviews in this study was primarily to begin codebook development and interview guide piloting for future research. The generalizability of the findings arose as another limitation, as qualitative data were only gathered at one institution. The generalizability of the study was adequate considering that the majority of medical schools in Nepal use the same curriculum models as the flagship programs, TU-IOM and KUSMS [64], though the findings cannot necessarily be generalized to students in rural medical programs.

In this study, we only conducted post-intervention assessments, so we were unable to compare the effects of the videos pre- and post-intervention or the long-term effects. Additional research is necessary to find long-term solutions for stigma reduction among medical students in LMICs, such as involving service users. Previous research has found the combination of educational and contact videos to be an effective intervention among medical students [26]. Future research could consider adding a fourth study arm with both didactic and service user testimonial videos to see if the combination of approaches can reinforce the information learned about mental illness and improve student attitudes.

## 5. Conclusions

In Nepal, there are not enough psychiatrists to meet the needs of people living with mental illness. Therefore, physicians from other disciplines need to be sensitive towards these issues so that people can receive quality physical health and mental health services. We demonstrated that didactic lectures and service user testimonials could be part of a much more extensive strategy to improve explicit attitudes regarding mental health during medical school. Contrary to expectations, including the psychosis service user testimonial video in addition to depression videos may have increased explicit stigma towards both depression and psychosis. Additional research is needed to evaluate the impact of bundling mental illnesses into training packages with and without service user involvement. Currently, service user involvement in mental health training of health providers is underutilized in LMICs [65]. As anti-stigma programs become more popular in LMICs, it will be important to consider how to best include service users in research to improve attitudes without causing harm. Incorporating service user narratives into training activities introduces the potential for introducing uncomfortable topics about mental illness, but it is also integral that health providers learn about the lived experiences of people with mental illnesses to enrich their understanding as practitioners [36]. Balancing the needs to respect those lived experiences and to improve attitudes is a challenge that must be addressed in future research. 

## Figures and Tables

**Table 1:** Content of didactic and service user testimonial videos (8 min videos).

Time ^1^ (m:ss)	Depression Videos ^2^		Psychosis Videos ^3^	
	Didactic Lecture Video	Service User Testimonial Video	Didactic Lecture Video	Service User Testimonial Video
0:00–0:59	Introduction to psychiatrist and mhGAP-IG module for depression	Introduction and family history of the service user living with depression	Introduction to psychiatrist and mhGAP-IG module for psychosis; acute versus chronic psychosis	Introduction to service user living with psychosis; description of symptom onset during pregnancy
1:00–1:59	Severity and signs of depression; symptoms of depression with examples	Father falling ill precipitating symptoms of depression, which are described in detail affecting daily activities	Continued acute versus chronic psychosis; symptoms of psychosis	Service user’s sister-in-law describes episodes of psychosis; service user describes suicide attempt
2:00–2:59	Continuation of symptoms; instructions for how to assess and diagnose depression	Service user takes mother to the health clinic, finds a handout with symptoms of depression, and continues to list symptoms of depression	Instructions for how to diagnose psychosis; description of three types of psychosis	Service user seeks medical treatment; reports that visiting a traditional healer did not help her symptoms
3:00–3:59	Overview of treatments: psychosocial counseling, psychoeducation, and medicine	Health worker speaks about the service user’s diagnosis of depression, comforts her, and referring the service user for counseling	Continued types of psychosis; responding to imminent suicide risk	Counselor describes symptoms of psychosis to make a diagnosis
4:00–4:59	Detailed explanation of psychosocial counseling and psychoeducation components	Counselor describes psychosocial counseling and psychoeducation skills she used with the service user	Three interventions: family involvement, psychoeducation, and counseling	Counselor describes therapy techniques; service user describes therapeutic activities she practices
5:00–5:59	Continued explanation of psychosocial counseling and psychoeducation	Service user recalls all of the tools she learned through counseling and psychoeducation	List of medications to treat psychosis; follow-up instructions	Counselor describes three types of psychosis and three types of interventions: family involvement, psychoeducation, and counseling
6:00–6:59	List of which medications to prescribe and how to prescribe them; follow-up procedures	Service user recovers without medication; she describes what medications are commonly prescribed for depression	Instructions for how to monitor patients with psychosis	List of medications to prescribe; service user describes recovery after she started medication; counselor describes follow-up procedure
7:00–7:45	Recovery description of how people living with depression can recover and return to normal activities; closing credits	Service user participates in mental health training to educate health workers about depression and sings her own song about mental illness; closing credits	Recovery description of how people living with psychosis can return to normal activities; closing credits	Service user resumes her regular activities at home, such as cooking, cleaning, and gardening; closing credits

^1^ Content of 8 min video is broken down into 60 s intervals. ^2^ Study 1 only included the depression videos. ^3^ Study 2 included depression and psychosis videos.

**Table 2:** Study 1 (depression only) participant demographic characteristics by arm.

Variables	Control: No Video (*n* = 31)	Didactic Video (*n* = 31)	Service User Video (*n* = 32)
Age			
Mean (SD)	21.03 (1.14)	21.19 (1.11)	21.00 (1.08)
Gender			
Male	23 (24%)	25 (27%)	23 (24%)
Female	8 (8%)	6 (6%)	9 (10%)
Class Level			
Second Year	14 (15%)	15 (16%)	16 (17%)
Third Year	17 (18%)	16 (17%)	16 (17%)
Personal experience or has family member/friend with mental illness			
Yes	18 (19%)	13 (14%)	13 (14%)
No	9 (10%)	14 (15%)	16 (17%)
Don’t Know	4 (4%)	4 (4%)	3 (3%)
Previous knowledge about mental illness from schooling			
Yes	10 (11%)	10 (11%)	6 (6%)
No	21 (22%)	21 (22%)	26 (28%)
Previous knowledge about mental illness outside of schooling			
Yes	18 (19%)	21 (22%)	22 (23%)
No	13 (14%)	10 (11%)	10 (11%)
Frequent Computer Users			
Yes	29 (33%)	28 (32%)	30 (34%)
No	2 (2%)	3 (3%)	2 (2%)
Medical specialty interest in psychiatry	2 (2%)	1 (1%)	0 (0%)

**Table 3:** Bivariate analyses of the effects of didactic and service user video interventions versus no video control on attitudes and depression knowledge.

Outcomes	M (SD)	β coef.	95% CI	*p*-Value
Explicit Attitude (SDS)				
Control: no video	39.10 (11.14)			
Didactic	33.32 (9.57)	−5.90	−10.95–−0.86	0.02 *
Service User	30.13 (9.16)	−8.85	−13.85–−3.84	0.001 *
Implicit Attitude (IAT) ^†^				
Harmfulness Attribute				
Control	0.15 (0.32)			
Didactic	0.07 (0.33)	−0.02	−0.11–0.06	0.59
Service User	0.04 (0.36)	−0.13	−0.30–0.04	0.13
Burdensomeness Attribute				
Control	0.20 (0.31)			
Didactic	0.10 (0.34)	−0.05	−0.13–0.03	0.25
Service User	0.17 (0.29)	−0.03	−0.19–0.13	0.70
Diagnosis Accuracy				
Control	0.77 (0.43)			
Didactic	0.84 (0.37)	0.21	−0.43–0.85	0.52
Service User	0.91 (0.30)	1.04	−0.42–2.49	0.16

Notes. Abbreviations: M, mean; SD, standard deviation; CI, confidence interval; SDS, Social Distance Scale; IAT, Implicit Association Test. ^†^ Sample *n* = 87 after excluding infrequent computer users. * Statistically significant at *p* < 0.05.

**Table 4:** Study 2 (depression and psychosis) participant demographic characteristics in each study condition (*n* = 213).

Variables	Control: No Video (*n* = 74)	Didactic Video (*n* = 70)	Service User (*n* = 69)
Age			
M (*SD*)	19.62 (0.95)	19.89 (1.21)	19.81 (1.19)
Institution			
TU-IOM	32 (15%)	34 (16%)	36 (17%)
KUSMS	42 (20%)	35 (16%)	34 (16%)
Gender			
Male	57 (27%)	55 (26%)	53 (25%)
Female	17 (8%)	15 (7)	16 (8%)
Class Level			
First Year	40 (19%)	35 (16%)	36 (17%)
Second Year	34 (16%)	35 (16%)	33 (15%)
Personal experience or has family member/friend with mental illness			
Yes	46 (22%)	41 (19%)	38 (18%)
No	22(10%)	11 (5%)	20 (9%)
Don’t Know	6 (3%)	18 (8%)	11 (5%)
Previous knowledge about mental illness from schooling			
Yes	58 (27%)	56 (26%)	49 (23%)
No	16 (8%)	14 (7%)	20 (9%)
Previous knowledge about mental illness outside of schooling			
Yes	62 (29%)	56 (26%)	48 (23%)
No	12 (6%)	14 (7%)	21 (10%)
Frequent Computer Users			
Yes	65 (31%)	60 (28%)	60 (28%)
No	9 (4%)	10 (5%)	9 (4%)
Medical specialty interest in			
Psychiatry	3 (1%)	2 (1%)	3 (1%)

Abbreviations: TU-IOM: Tribhuvan University—Institute of Medicine; KUSMS: Kathmandu University School of Medical Sciences.

**Table 5:** Effects of didactic and service user video interventions versus no video (control group) on negative stigmatizing attitudes and increased knowledge about mental illnesses.

Outcomes	M (SD)	β coef. (95%CI)	*p*-Value
Explicit Attitude (SDS)			
Control	41.47 (9.49)		
Didactic	41.01 (9.43)	−0.45 (−3.74–2.83)	0.79
Service User	44.39 (10.97)	2.74 (−0.55–6.02)	0.10
Unsafe around people who have mental illness			
Depression Vignette			
Control	3.59 (1.07)		
Didactic	3.57 (1.06)	0.01 (−0.35–0.36)	0.97
Service User	3.13 (1.10)	−0.43 (−0.78–(−0.07))	0.02 *
Psychosis Vignette			
Control	2.81 (1.02)		
Didactic	2.74 (1.10)	−0.09 (−0.43–0.26)	0.61
Service User	2.52 (1.02)	−0.23 (−0.58–(−0.11))	0.18
Potentially violent towards others			
Depression Vignette			
Control	3.32 (1.06)		
Didactic	3.21 (1.05)	−0.07 (−0.42–0.27)	0.67
Service User	2.81 (1.06)	−0.50 (−0.84–(−0.15))	0.005 *
Psychosis Vignette			
Control	2.51 (0.85)		
Didactic	2.44 (0.91)	−0.10 (−0.39–0.19)	0.49
Service User	2.13 (0.84)	−0.33 (−0.62–(−0.05))	0.02 ^△^
Implicit Attitude (IAT) ^⟐^			
Harmfulness Attribute			
Control	0.01 (0.31)		
Didactic	−0.004 (0.30)	−0.04 (−0.15–0.08)	0.54
Service User	0.02 (0.33)	−0.01 (−0.10–0.13)	0.81
Burdensomeness Attribute			
Control	0.06 (0.29)		
Didactic	0.09 (0.34)	0.03 (−0.09–0.15)	0.61
Service User	0.11 (0.35)	0.05 (−0.06–0.17)	0.36
Diagnostic Accuracy of Depression and Psychosis			
Control	0.36 (0.48)		
Didactic	0.60 (0.49)	1.02 (0.34–1.69)	0.003 *
Service User	0.45 (0.50)	0.37 (−0.30–1.04)	0.28
Treatment Accuracy			
Depression Vignette			
Control	2.03 (1.18)		
Didactic	2.67 (1.21)	0.68 (0.29–1.07)	0.001 *
Service User	2.54 (1.16)	0.48 (0.09–0.87)	0.02 *
Psychosis Vignette			
Control	2.26 (1.14)		
Didactic	2.87 (1.09)	0.63 (0.25–1.01)	0.001 *
Service User	2.72 (1.24)	0.51 (0.14–0.89)	0.008 *

Notes. Abbreviations: M, mean; CI, confidence interval; IAT, Implicit Association Test; SDS, Social Distance Scale; *SD*, standard deviation. ^△^ Entire linear regression model is not statistically significant (*F*_2,210_ = 2.73, *p* = 0.07, *R*^2^ = 0.04). ^⟐^ *n* = 182 after excluding infrequent computer users and missing data. * Statistically significant at *p* < 0.05.

**Table 6:** Associations between explicit and implicit attitudes towards mental illness and knowledge to diagnose and treat depression and psychosis (*n* = 213).

Outcomes	Social Distance Scale (SDS)	Implicit Attitude Test—Harmfulness	Implicit Attitude Test—Burdensomeness
Implicit Attitude (IAT) ^△^: Harmfulness Attribute	*r* = −0.01 *p* = 0.86		
Implicit Attitude (IAT) ^△^: Burdensomeness Attribute	*r* = 0.09 *p* = 0.21	*r* = 0.27 ^†^ *p* < 0.001 **	
Explicit Attitude (Uncomfortable in public near someone with depression)	*r* = −0.23 *p* < 0.001 **	*r* < 0.01 *p* = 0.96	*r* = −0.09 *p* = 0.20
Explicit Attitude (Uncomfortable in public near someone with psychosis)	*r* = −0.30 *p* < 0.001 **	*r* = −0.03 *p* = 0.66	*r* = −0.19 *p* = 0.007 *
Explicit Attitude (People with depression are violent)	*r* = −0.10 *p* = 0.13	*r* = −0.08 *p* = 0.25	*r* = −0.05 *p* = 0.49
Explicit Attitude (People with psychosis are violent)	*r* = −0.18 *p* = 0.008 *	*r* = −0.11 *p* = 0.13	*r* = −0.12 *p* = 0.11
Knowledge (Accurately diagnosed depression and psychosis)	*r* = −0.08 *p* = 0.23	*r* = 0.13 *p* = 0.09	*r* = 0.08 *p* = 0.30
Knowledge (Treatment accuracy for depression)	*r* = 0.03 *p* = 0.68	*r* = −0.11 *p* = 0.12	*r* = 0.07 *p* = 0.32
Knowledge (Treatment accuracy for psychosis)	*r* = −0.06 *p* = 0.42	*r* = −0.04 *p* = 0.56	*r* = 0.01 *p* = 0.84

Notes. Abbreviations: SDS, Social Distance Scale; IAT, Implicit Association Test. ^△^ *n* = 182 and ^†^ *n* = 180 after excluding infrequent computer users and missing data. * Statistically significant at *p* < 0.05. ** Statistically significant at *p* < 0.001.

**Table 7:** Personal experience with mental illness adjusted model (*n* = 178).

Explicit Attitude (SDS)	M (SD)	β Coef. (95%CI)	*p*-Value
Control	40.81 (9.11)	--	--
Didactic	41.69 (10.19)	0.19 (−3.38–3.77)	0.91
Service User	45.53 (10.68)	4.44 (1.01–7.87)	0.01 *

Notes. Abbreviations: SDS, Social Distance Scale. * Statistically significant at *p* < 0.05.

## Data Availability

Data are available from the author upon request.
